# Expression Patterns of Tumor Markers in Liver Transplant Recipients Showing Complete Pathological Response of Hepatocellular Carcinoma

**DOI:** 10.3390/jcm11195897

**Published:** 2022-10-06

**Authors:** Min-Jae Kim, Woo-Hyoung Kang, Shin Hwang, Chul-Soo Ahn, Deok-Bog Moon, Tae-Yong Ha, Gi-Won Song, Dong-Hwan Jung, Gil-Chun Park

**Affiliations:** Department of Surgery, Asan Medical Center, University of Ulsan College of Medicine, 88 Olympic-ro 43-gil, Songpa-gu, Seoul 05505, Korea

**Keywords:** locoregional treatment, treatment response, pathologic response, radiological response, liver transplantation

## Abstract

Complete pathological response (CPR) is achieved with various pretransplant locoregional treatments for hepatocellular carcinoma (HCC). This study aimed to investigate pretransplant expression of HCC tumor markers in liver transplantation (LT) recipients showing CPR. For the CPR group, 166 patients were selected from a single-institution LT database. Two control groups of 332 patients without HCC and 184 patients with partial pathological response (PPR) were also selected. The model for end-stage liver disease score in the CPR group was 11.5 ± 7.7. The number of transcatheter arterial chemoembolization sessions before LT was one in 68 patients (14.0%), two in 38 patients (22.9%), and three or more in 60 patients (36.1%). A solitary non-viable tumor was identified in 120 (86.4%) of the explant livers and the largest tumor size was 2.4 ± 1.3 cm. Living-donor and deceased-donor LTs were performed in 152 (91.6%) and 14 (8.4%) patients, respectively. The median levels of α-fetoprotein (AFP) and protein induced by Vitamin K absence or antagonist-II (PIVKA-II) measured within two weeks before LT were 4.2 ng/mL and 20 mAU/mL, respectively. These tumor marker levels were comparable to those in the no-HCC control group, but much lower than those in the PPR group (*p* < 0.001). Receiver operating characteristic curve analysis of AFP and PIVKA-II showed no definite cutoff values for CPR in the cohort of CPR and no-HCC patients, but significant cutoffs of 6.5 ng/mL for AFP and 29 mAU/mL for PIVKA-II were obtained in the cohort of CPR and PPR patients. The 1-, 3- and 5-year HCC recurrence and overall patient survival rates of the CPR group were 5.1% and 93.3%, 7.6% and 89.6%, and 7.6% and 89.6%, respectively. These tumor recurrence rates were much lower than those in the PPR group (*p* < 0.001). In conclusion, the present study results suggest that normalizing AFP and PIVKA-II after locoregional treatment is indicative of CPR. However, some CPR patients showed high expression of tumor markers; thus, pretransplant values of HCC tumor markers should be interpreted with caution.

## 1. Introduction

Hepatocellular carcinoma (HCC) is the fifth most common malignancy and one of the leading causes of cancer-related patient deaths worldwide [1]. Liver transplantation (LT) has been regarded as a definitive treatment for patients with liver cirrhosis and early-stage HCC. However, patients with HCC beyond this stage may benefit from LT if their tumors are successfully downstaged [2,3]. Notably, LT is limitedly indicated by the high waitlist dropout incidence due to long waiting periods and limited organ availability. Consequently, bridging or salvage therapy is recommended for patients with expected long wait times. Various locoregional treatments (LRTs) have been performed in HCC patients for cure or downstaging before LT [3,4]. Transcatheter arterial chemoembolization (TACE) is the most common initial HCC treatment [5,6]. LRTs, including TACE, can achieve complete pathological response (CPR), which is confirmed through pathological evaluation of the explant liver. CPR presents a loss of viable tumor cells, thus being considered a reliable indicator of favorable posttransplant prognosis [7,8,9]. Pretransplant prediction of CPR is still a challenge because of the incomplete reliability of imaging findings and variable expression of tumor markers in patients with advanced liver cirrhosis. Therefore, this study aimed to investigate the pretransplant expression patterns of HCC tumor markers in LT recipients who achieved CPR of HCC at the explant livers.

## 2. Patients and Methods

### 2.1. Study Design

This study is a retrospective observational study with one study group (CPR group) and two control groups, one without HCC and one with partial pathological response (PPR) of HCC.

### 2.2. Selection of Study Group Patients with CPR of HCC

Our institutional LT database was searched to identify adult patients with HCC who had undergone primary LT between January 2006 and December 2015. The inclusion criteria were CPR of HCC in the explant livers, defined as the absence of viable tumor cells in all tumor nodules (tumor necrosis ≥ 99%). The exclusion criteria were no history of pretransplant LRT, salvage LT after initial hepatic resection, liver pathology other than HCC such as combined HCC-cholangiocarcinoma, and no measurement of α-fetoprotein (AFP) and protein induced by Vitamin K absence or antagonist-II (PIVKA-II) within two weeks before LT. After patient exclusion through the base of the clinicopathological profiles, we finally selected 166 patients showing LRT-induced CPR of HCC in the explant livers. Patient medical records were retrospectively reviewed. These study patients were followed up until December 2020 through medical record review and with the assistance of the National Health Insurance Service.

### 2.3. Pretransplant Diagnosis for HCC

Patients with chronic liver disease were followed up for surveillance of HCC according to the Korean Association for the Study of the Liver guidelines [10,11]. Regular pretransplant evaluation for HCC included magnetic resonance imaging, abdomen and chest computed tomography, and fluorodeoxyglucose positron-emission tomography. The details of the preoperative evaluation process were previously described [12,13]. The upper normal values of AFP and PIVKA-II in our institution were 7.5 ng/mL and 40 mAU/mL, respectively.

### 2.4. Preoperative Locoregional Treatment and Pathological Assessment

Preoperative LRTs, including TACE, have been performed as a curative treatment, palliative cytoreduction therapy, downstaging treatment, and response tests for LT suitability, or their combinations [13]. In the CPR group, all 166 patients underwent TACE and the majority of them received multiple sessions of TACE prior to LT. In addition to TACE, radiofrequency ablation and external beam radiotherapy were performed before or after TACE. CPR was defined as extensive necrosis of ≥99% of the total tumor volume, indicating no viable tumor cells in any nodule.

### 2.5. Selection of Control Group Patients without HCC

The CPR group showed lower expression of tumor markers after LRT before LT, but no cutoff values were proposed to predict CPR in the literature. To perform a statistical comparison of tumor markers between the CPR study group and the no-HCC group, a control group without HCC was selected among 2500 patients who underwent LT during the study period through propensity score matching. An optimal propensity score matching method was used [14], and the matching parameters were age, sex, model for end-stage liver disease (MELD) score, background liver disease, and LT type. Through a 1:2 matching, 332 LT recipients who underwent living-donor LT or deceased-donor LT during the study period were selected as the control group. Between the CPR and control groups, baseline patient characteristics were very similar, and the only differences were pretransplant diagnosis of HCC and performance of LRT in the CPR group.

### 2.6. Selection of Control Group Patients with Partial Pathological Response of HCC

The patients with PPR following LRT were selected as a control group showing partial response. The PPR control group patients were selected by searching our institutional LT database. The inclusion criteria were extensive PPR of HCC at the explant livers, which were arbitrarily defined as extensive tumor necrosis between 70% and 98% in the pathology report. These HCC lesions were regarded as viable tumors. The patients who had CPR of one nodule and PPR of another were considered as the PPR group. The exclusion criteria were the same for the CPR group. We finally selected 184 patients showing LRT-induced PPR of HCC at the explant livers.

### 2.7. Statistical Analysis

Numerical data are presented as mean and standard deviation or median with 25–75 percentiles. Continuous variables were compared using the Student’s *t*-test and analysis of variance. Survival curves were generated using the Kaplan-Meier method. Receiver operating characteristic (ROC) curve analysis with the Youden J index was performed to determine cutoff values of tumor markers. A *p*-value < 0.05 was considered to be statistically significant. Propensity score matching was performed using Stata version 15 (StataCorp, College Station, TX, USA). All statistical analyses were performed using SPSS version 22 (IBM, New York, NY, USA) and MedCalc version 20.010 (MedCalc, Ostend, Belgium).

## 3. Results

### 3.1. Patient Demographics

The demographics and tumor characteristics of the CPR study, no-HCC control, and PPR control group patients are summarized in Table 1. In the CPR study group, there were 148 cases (89.2%) of hepatitis B virus-associated liver cirrhosis. The MELD score was 11.5 ± 7.7. The number of pretransplant TACE sessions was one in 68 patients (14.0%), two in 38 patients (22.9%), and three or more in 60 patients (36.1%). According to the pathology report findings of the explant livers, the number of patients with solitary non-viable tumors was 120 (86.4%), and the size of the largest non-viable tumor was 2.4 ± 1.3 cm. Living-donor and deceased-donor LTs were performed in 152 (91.6%) and 14 (8.4%) patients, respectively.

The non-HCC control group was selected after matching for sex, MELD score, background liver disease, and LT type; thus, their clinical profiles were similar to those of the CPR study group (Table 1).

The PPR control group showed similar demographic profiles to the CPR study group, but higher expression of AFP and PIVKA-II, larger maximal tumor size, and frequent tumor multiplicity were identified.

### 3.2. Expression Patterns of HCC Tumor Markers in the CPR Study Group

**Figure 1:**
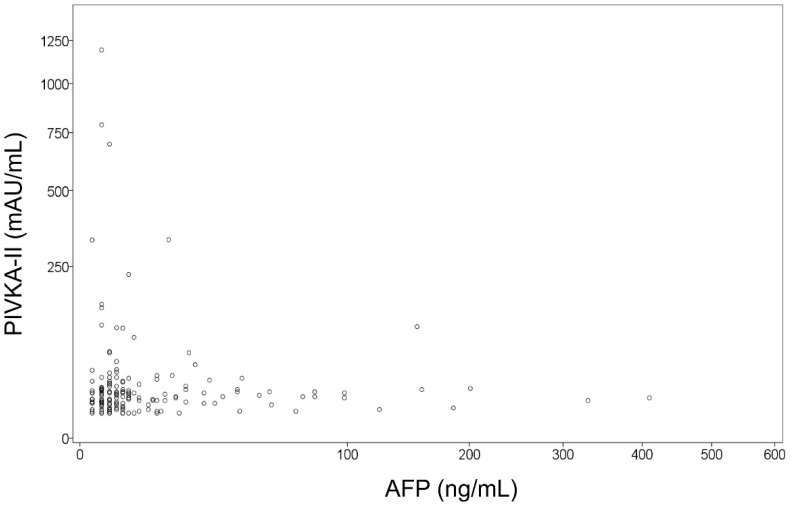
Scatter plot showing the association between α-fetoprotein (AFP) and protein induced by Vitamin K absence or antagonist-II (PIVKA-II) expression in patients with hepatocellular carcinoma of complete pathological response.

The expression patterns of AFP and PIVKA-II measured within two weeks before LT are depicted in Figure 1. This tumor marker measurement was time-matched with the tumor status of CPR. Expression of AFP showed a median value of 4.2 ng/mL (25–75 percentiles: 2.2–12.0). Expression of PIVKA-II showed a median value of 20 mAU/mL (25–75 percentiles: 15–28; Figure 2). With the application of institutional cutoff values, normal AFP and PIVKA-II levels were observed in 115 (69.3%) and 144 (86.8%) patients, respectively.

### 3.3. ROC Curve Analysis of Tumor Markers for CPR

**Figure 3:**
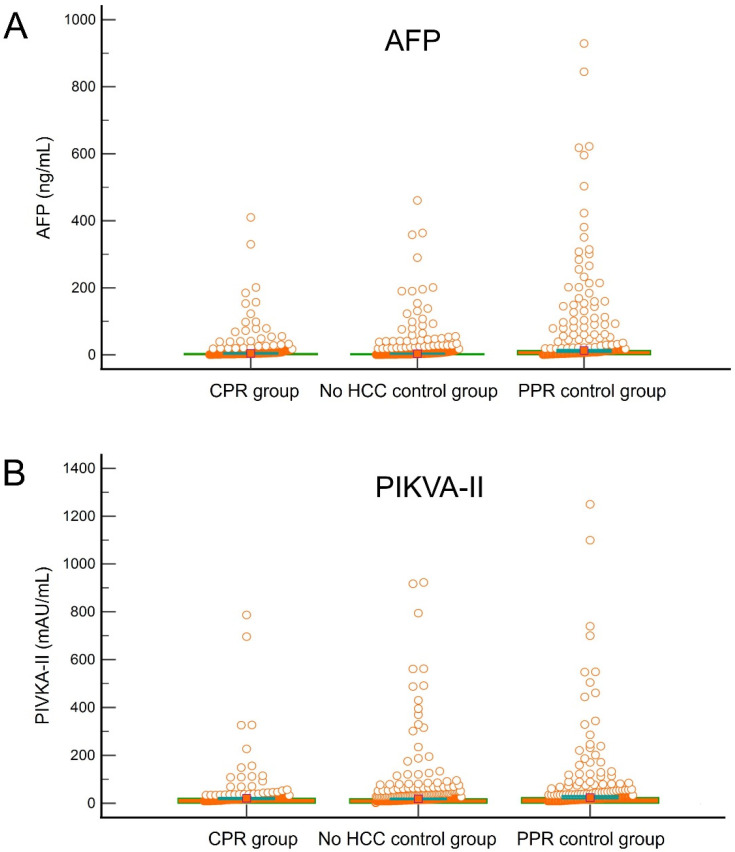
Comparison of α-fetoprotein (AFP; (**A**)) and protein induced by Vitamin K absence or antagonist-II (PIVKA-II; (**B**)) expression between the study patients with hepatocellular carcinoma of complete pathological response (CPR), control patients without hepatocellular carcinoma and partial pathological response (PPR) control group. Bars indicate the median values.

**Figure 4:**
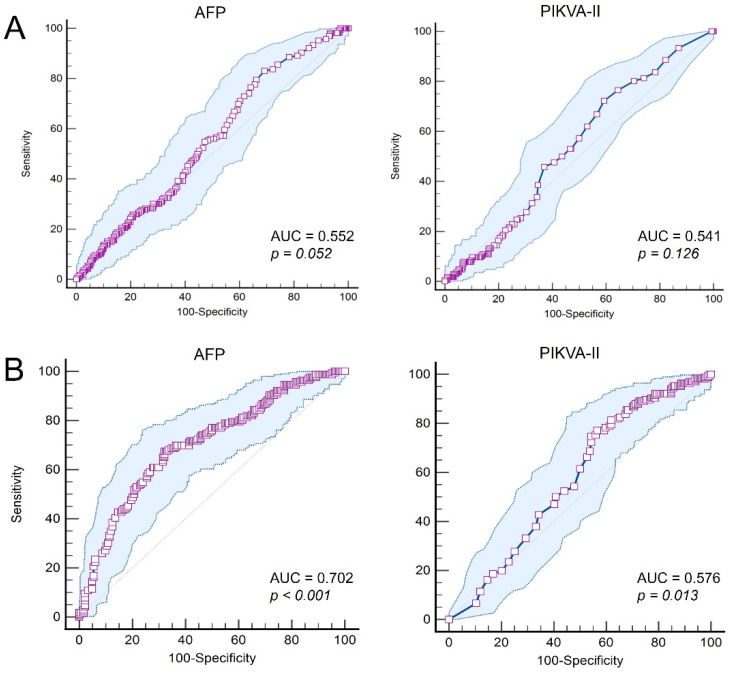
Receiver operating characteristic curve analysis of α-fetoprotein (AFP) and protein-induced by Vitamin K absence or antagonist-II (PIVKA-II) for predicting complete pathological response in the cohort of complete pathological response study patients and no-hepatocellular carcinoma patients (**A**) and the cohort of complete pathological response study patients and partial pathological response control patients (**B**). AUC, area under the curve.

In the study cohort including 166 HCC patients with CPR and 332 control patients without HCC, the median AFP was 4.2 ng/mL (25–75 percentiles: 2.2–12.0) and 4.0 ng/mL (25–75 percentiles: 2.1–10.0), respectively (*p* = 0.596), and the median PIVKA-II was 20 mAU/mL (25–75 percentiles: 15–28) and 19 mAU/mL (25–75 percentiles: 12–31) respectively (*p* = 0.981; Figure 3). ROC curve analysis of AFP and PIVKA-II showed no definite cutoff values for CPR (Figure 4A). The ROC area under the curve (AUC) was 0.552 (95% confidence interval: 0.507–0.596; *p* = 0.052) for AFP and 0.541 (95% confidence interval: 0.496–0.585; *p* = 0.126) for PIVKA-II.

In the study cohort, including 166 study HCC patients with CPR and 184 control patients with PPR, the PPR group showed a median AFP of 12.4 ng/mL (25–75 percentiles: 5.0–56.3) (*p* < 0.001 compared with CPR) and a median PIVKA-II of 23 (25–75 percentiles: 15–53) (*p* < 0.001 compared with CPR; Figure 3). ROC curve analysis of AFP and PIVKA-II showed statistically significant cutoff values for CPR (Figure 4B). The ROC AUC was 0.702 (95% confidence interval: 0.651–0.749; *p* < 0.001). An AFP cutoff of 6.5 ng/mL showed a sensitivity of 67.5% and a specificity of 67.9%, with a Youden index J of 0.354. The ROC AUC for PIVKA-II was 0.576 (95% confidence interval: 0.522–0.628; *p* = 0.013). A PIVKA-II cutoff of 29 mAU/mL showed a sensitivity of 77.1% and a specificity of 43.5%, with a Youden index J of 0.206.

### 3.4. Tumor Recurrence and Overall Patient Survival after Liver Transplantation

**Figure 5:**
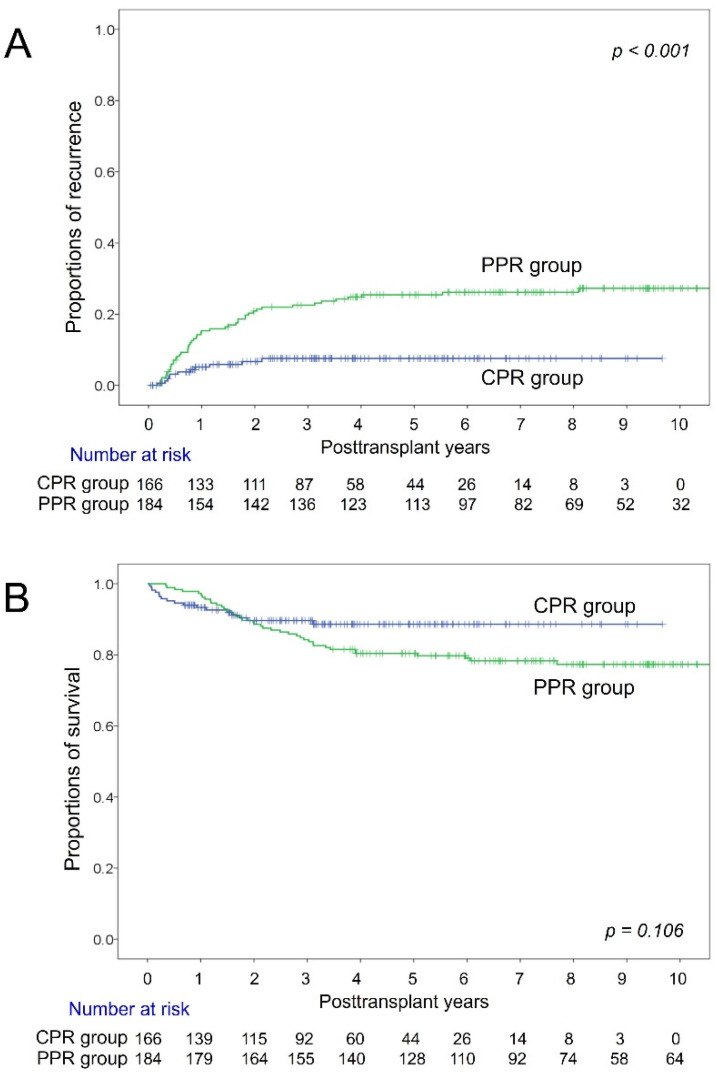
Kaplan-Meier curves of tumor recurrence (**A**) and overall patient survival (**B**) in liver transplant recipients showing hepatocellular carcinoma with the complete pathological response (CPR) and partial pathological response (PPR).

During the follow-up period of up to 10 years, tumor recurrence occurred in 11 (6.6%) patients of the CPR group; thus the 1-, 3- and 5-year tumor recurrence rates were 5.1%, 7.6%, and 7.6%, respectively. All-cause death occurred in 17 (10.2%) patients (HCC recurrence-associated death in 8, infection in 4, vascular complication in 3, chronic rejection in 1, and cardiovascular disease in 1); thus the 1-year, 3-year, and 5-year overall patient survival rates were 93.3%, 89.6%, and 88.6%, respectively (Figure 5).

In the PPR group, the 1-year, 3-year, and 5-year tumor recurrence and overall patient survival rates were 15.3% and 97.3%, 20.9% and 88.6%, and 25.4% and 80.4%, respectively (*p* < 0.001 for tumor recurrence and *p* = 0.106 for patient survival; Figure 5).

### 3.5. Pretransplant Tumor Marker Levels and Posttransplant Tumor Recurrence in the CPR Group

In the CPR group, 11 patients showing tumor recurrence had the median pretransplant levels of AFP of 4.4 ng/mL (25–75 percentiles: 2.4–5.9) and PIVKA-II of 23 mAU/mL (25–75 percentiles: 18–27), which were not different from AFP of 4.2 ng/mL (25–75 percentiles: 2.3–12.1; *p* = 0.674) and PIVKA-II of 21 mAU/mL (25–75 percentiles: 14–28; *p* = 0.563) in the other 155 patients without tumor recurrence.

The sites of initial tumor recurrence were intrahepatic in two patients, extrahepatic in eight patients, and concurrent intra- and extrahepatic in one patient. The sites of extrahepatic recurrence were lung in four patients, bone in two patients, and intraperitoneal recurrence in three patients.

In 13 patients showing pretransplant level of AFP > 100 ng/mL or PIVKA-II > 200 mAU/mL, only 1 patient showed tumor recurrence.

## 4. Discussion

LRT-associated CPR is considered an indicator of improved prognosis in HCC patients [7,8,9]. The occurrence of CPR after LRT is a matter of concern since such CPR is related to a favorable post-treatment prognosis, as shown in the present study. Complete radiologic response (CRR), which is evaluated using the modified Response Evaluation Criteria in Solid Tumor (mRECIST) [15], and post-LRT normalization of HCC tumor markers are important clues for pretransplant prediction of CPR.

In our previous study, the incidence of CPR was 233 (31.6%) of 738 LT recipients [7]. In a study with 67 patients who underwent LT for HCCs < 5 cm, CRR was identified in 44 patients (65.7%) with 71 nodules [16]. Another study revealed that the incidence of CPR was estimated as 47.9% for patients with a single tumor, with maximal tumor size < 30 mm and preoperative AFP < 100 ng/mL [9]. Although CRR was achieved after LRT, a non-negligible proportion of HCC patients had to receive LT because of advanced liver cirrhosis. The multiple sessions of TACE were associated with a higher incidence of CPR in the LT recipients [7].

Because treatment response to TACE is variable in patients with HCC, TACE has been repeatedly performed to raise treatment response. In a study using explant livers [17], near-complete tumor necrosis was induced through extensive lipiodol accumulation within the tumor. A Korean study reported that overall patient survival was related to the initial and the best TACE response [18]. Meanwhile, another study suggested that the best radiological response to TACE was not significantly associated with improved outcomes [19]. In a Korean study with 890 patients with TACE-induced CRR, the median AFP level after achieving CRR was 6.36 ng/mL, and high AFP levels at CRR (>20 ng/mL) were an independent risk factor for high tumor recurrence and low patient survival [20]. Therefore, CRR combined with normalization of tumor markers following initial or repeated TACE is a viable reason for delaying LT in patients with compensated liver cirrhosis and early-stage HCC.

Effective treatment of posttransplant HCC recurrence is difficult, and therefore prevention of tumor recurrence is important. TACE has been used in real-world practice as a treatment with curative intent, for downstaging effects, or to observe the treatment response for selection of eligible LT candidates. A few studies have suggested that the prognostic advantage of pretransplant TACE is not definite since it induces only a downstaging effect [6,21,22]. In addition to TACE, other LRTs, such as radiofrequency ablation and external beam radiotherapy, have been frequently performed to increase treatment response. LRT-induced CPR can provide a significant prognostic benefit because the tumor load can be theoretically minimized. CPR, per se, means a marked reduction in tumor cell load; thus, it would be beneficial to decrease posttransplant tumor recurrence, as shown in the present study [8,9]. PPR means extensive tumor necrosis, but viable tumor portions are left in the liver. The CPR group showed much lower tumor recurrence rates compared with the PPR group in the present study.

In the present study, the median AFP level was 4.2 ng/mL and 4.0 ng/mL in the study patients with CPR and control patients without HCC, respectively. The median PIVKA-II level was 20 mAU/mL and 19 mAU/mL in the CPR study patients and control no-HCC patients, respectively. These results suggest that tumor marker expression in CPR patients is comparable to that in patients without HCC. In contrast, the PPR group showed higher expression of AFP and PIVKA-II than the CPR group and no-HCC group. A significant decrease in tumor markers is associated with extensive tumor necrosis. The expression patterns of AFP and PIVKA-II are widely variable in HCC patients [23,24,25]. These tumor markers can be highly expressed in patients even with no HCC, especially those with advanced liver cirrhosis. Consequently, the threshold of AFP and PIVKA-II for diagnosis of HCC in patients with advanced liver cirrhosis would be higher than in HCC patients with normal liver or chronic hepatitis [26]. In our precedent study with 1595 LT recipients with HCC and 1496 LT recipients without HCC, the median AFP level was 9.0 ng/mL in the HCC group and 4.0 ng/mL in the non-HCC group (*p* < 0.001). The median PIVKA-II level was 25 mAU/mL in the HCC group and 23 mAU/mL in the non-HCC group (*p* = 0.274). Particularly, when confined to patients with alcoholic liver disease, the median PIVKA-II level was 54 mAU/mL in the HCC group and 48 mAU/mL in the non-HCC group (*p* = 0.456) [27].

It is reported that serum AFP (>15 or 20 ng/mL) as a screening test for HCC had a sensitivity between 39% and 64%, a specificity between 76% and 91%, and a positive predictive value between 9% and 33% [28,29]. A case–control study of 340 patients with liver cirrhosis showed that AFP levels > 20 ng/mL had a sensitivity of 60% and a specificity of 91% in the diagnosis of HCC. With the application of this cutoff value, 40% of the HCC cases would have been missed from diagnosis [30].

In the present study, ROC curve analysis of AFP and PIVKA-II showed no noticeable cutoff values for CPR in the combined cohort of CPR and no-HCC patients. In contrast, in the combined cohort of CPR and PPR patients, significant cutoff values for CPR were identified. An AFP cutoff of 6.5 ng/mL showed a sensitivity of 67.5% and a specificity of 67.9%. A PIVKA-II cutoff of 29 mAU/mL showed a sensitivity of 77.1% and a specificity of 43.5%. These cutoff values indicate the normalization of tumor markers. HCC tumor markers are still highly expressed in PPR with 70–98% tumor necrosis, but the majority of CPR patients showed normalization of tumor markers.

In the present study, 11 patients showing tumor recurrence had median pretransplant levels of AFP 4.4 ng/mL and PIVKA-II 23 mAU/mL, which were not different from those in the other 155 patients without tumor recurrence. In 13 patients showing pretransplant levels of AFP > 100 ng/mL or PIVKA-II > 200 mAU/mL, only 1 patient showed tumor recurrence. These results indicate that the pretransplant level of AFP and PIVKA-II in patients showing CPR of HCC is not associated with posttransplant tumor recurrence.

Meanwhile, in patients with viable HCC, the pretransplant level of tumor markers is known to be associated with posttransplant prognosis. A Japanese study reported that worse disease-free and overall patient survival was correlated with the presence of at least two of the following factors: beyond the Tokyo criteria (≤5 tumors with each tumor ≤ 5 cm), AFP > 250 ng/mL, and PIVKA-II DCP > 450 mAU/mL (>450 ng/mL) [31]. High clinical correlation was achieved in these criteria through morphometric information of HCC and two tumor markers, but these criteria can be applicable only to patients with viable HCCs, but not to those with CPR of HCC.

AFP has been accepted as a reliable biomarker worldwide regarding the diagnosis and prognosis of HCC. In contrast, the measurement of PIVKA-II has been routinely performed only in some Asian countries, especially including Japan and Korea. We have measured PIVKA-II as a routine test of HCC for more than 20 years. On the other hand, in China, a large-scale multicenter study, which was launched in 2012, proved the diagnostic role of PIVKA-II [32]. Thereafter, PIVKA-II was included as one of the diagnostic markers for HCC in China [33]. In addition, a Japanese study also suggested that PIVKA-II ≥ 300 mAU/mL was an independent predictor of tumor recurrence following living-donor LT [34]. Currently, the pretransplant levels of AFP and PIVKA-II alone and their combination have been generally regarded as diagnostic and prognostic biomarkers of HCC in the field of LT [25,31,34].

This study has some limitations of note. It is a single-center retrospective study in an HBV-endemic country, indicating the necessity to assess the prognostic effect of CPR in various patients with other background liver diseases. Second, we did not include the serial changes in AFP and PIVKA-II after LRT or radiological response findings before and after LRT.

## 5. Conclusions

The results of the present study suggest that serum levels of AFP and PIVKA-II are normalized in the majority of patients with CPR of HCC following LRT, which was comparable to those in patients without HCC. Thus, normalization of tumor makers is indicative of CPR. However, some CPR patients showed high expression of tumor markers; thus, pretransplant values of HCC tumor markers should be interpreted with caution.

## Figures and Tables

**Figure 2:**
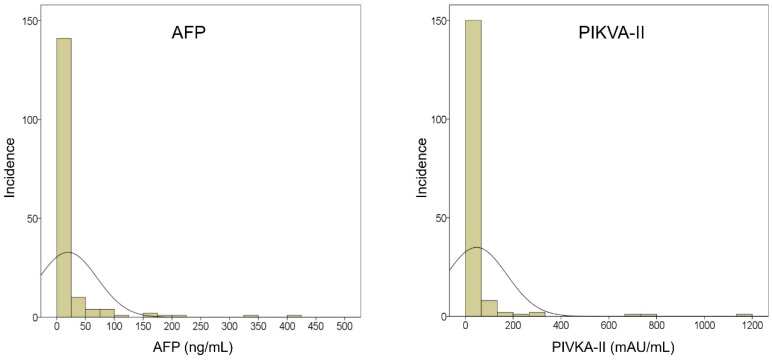
Distribution of α-fetoprotein (AFP) and protein induced by Vitamin K absence or antagonist-II (PIVKA-II) expression measured within two weeks before transplantation in patients with hepatocellular carcinoma of complete pathological response.

**Table 1:** Comparison of demographic and tumor characteristics profiles of the CPR study group, no-HCC control group, and PPR control group patients.

Group		Study CPR Group	No-HCC Control Group	PPR Control Group	*p*-Value	
Case number		166	332	184	CPR vs. No-HCC	CPR vs. PPR
Age (years)		54.0 ± 7.1	55.1 ± 7.8	53.6 ± 6.0	0.151	0.381
Gender (Male/Female) (n)		141/25	280/52	163/21	0.861	0.313
Background liver disease (n)					0.520	0.994
	HBV	148	302	164		
	HCV	11	20	12		
	ALD	4	0	6		
	Others	3	10	2		
Pretransplant blood laboratory profiles (mean ± SD)						
	Albumin (g/dL)	3.0 ± 0.6	2.9 ± 0.7	3.1 ± 0.7	0.137	0.271
	AST (IU/L)	58.7 ± 55.8	55.7 ± 62.5	52.8 ± 36.2	0.622	0.128
	ALT (IU/L)	41.4 ± 35.8	37.1 ± 42.5	37.6 ± 27.2	0.374	0.385
	Total bilirubin (mg/dL)	2.9 ± 6.6	4.8 ± 8.2	3.1 ± 0.7	0.014	0.179
	Platelet count (103/μL)	67.8 ± 47.3	54.2 ± 55.7	72.6 ± 63.1	0.011	0.191
	Prothrombin time (INR)	1.35 ± 0.53	1.43 ± 0.61	1.34 ± 0.38	0.174	0.821
AFP (ng/mL) at LT					0.596	<0.001
	Mean ± SD	19.6 ± 50.4	17.1 ± 48.8	67.9 ± 141.6		
	Median (25–75 percentiles)	4.0 (2.1–12.0)	4.0 (2.1–10.0)	12.4 (5.0–56.3)		
	7.5 ng/mL or lower (n, %)	115 (69.3%)	148/82	67 (36.4%)		
PIVKA-II (mAU/mL) at LT					0.981	<0.001
	Mean ± SD	46.7 ± 126.2	46.6 ± 109.7	77.4 ± 164.4		
	Median (25–75 percentiles)	20 (15–28)	19 (12–31)	23 (15–53)		
	40 mAU/m or lower (n, %)	144 (86.8%)	136/41	126 (68.5%)		
MELD score *		11.5 ± 7.7	12.8 ± 6.9	11.8 ± 8.1	0.078	0.487
TACE session (n)			NA		NA	0.066
	Single	68	NA	58		
	Two	38	NA	37		
	≥3	60	NA	89		
Other locoregional treatment (n)					NA	0.718
	Radiofrequency ablation	32	NA	41		
	External beam radiotherapy	12	NA	13		
Type of liver transplantation (n)					0.365	0.632
	LDLT	152	206	171		
	DDLT	14	26	13		
Maximal tumor diameter (cm)		2.4 ± 1.3	NA	3.0 ± 1.7	NA	<0.001
Tumor number (n)					NA	<0.001
	Single	120	NA	65		
	Two	27	NA	52		
	≥3	19	NA	67		
Macrovascular invasion (n)		NA	NA	10	NA	NA
Microvascular invasion (n)		NA	NA	29	NA	NA

*, Calculated within 1 week before LT. CPR, complete pathological response; PPR, partial pathological response; HCC, hepatocellular carcinoma; LT, liver transplantation; HBV, hepatitis B virus; HCV, hepatitis C virus; ALD, alcoholic liver disease; AST, aspartate aminotransferase; ALT, alanine aminotransferase; AFP, α-fetoprotein; PIVKA-II, proteins induced by vitamin K antagonist or absence-II; MELD, a model for end-stage liver disease; TACE, transcatheter arterial chemoembolization; LDLT, living-donor liver transplantation; DDLT, deceased-donor liver transplantation; NA, not available.

## Data Availability

The data underlying this article will be shared upon reasonable request to the corresponding author.
